# Improving the Quality of Life of Cancer Survivors in School: Consensus Recommendations Using a Delphi Study

**DOI:** 10.3390/children8111021

**Published:** 2021-11-07

**Authors:** Santiago Galán, Catarina Tomé-Pires, Rubén Roy, Elena Castarlenas, Mélanie Racine, Mark P. Jensen, Jordi Miró

**Affiliations:** 1 Universitat Rovira i Virgili, Unit for the Study and Treatment of Pain—ALGOS, Research Center for Behavior Assessment (CRAMC), Chair in Pediatric Pain Universitat Rovira i Virgili-Fudación Grünen-thal, Department of Psychology, 43007 Tarragona, Catalonia, Spain; sgalanortega@gmail.com (S.G.); jamnista@gmail.com (C.T.-P.); ruben.roy@urv.cat (R.R.); elena.castarlenas@urv.cat (E.C.); 2 Institut d’Investigació Sanitària Pere Virgili, 43007 Tarragona, Catalonia, Spain; 3 Clinical and Neurological Sciences Department, Schulich School of Medicine & Dentistry, Western University, London, ON J3Y 7H7, Canada; research@melanieracine.com; 4 Department of Rehabilitation Medicine, University of Washington, Seattle, WA 98104, USA; mjensen@uw.edu

**Keywords:** needs, resources, teacher, student cancer survivor, re-entry process, Delphi method

## Abstract

Successful school re-entry is important for children following cancer treatment. However, this process is a challenge for teachers. Objectives: To identify (1) the difficulties and needs that teachers have in helping youth cancer survivors be successful in school, (2) the most effective resources that teachers are currently using for helping them, and (3) the ideal contents for a program that could help teachers in this area. Methods: Twenty-eight teachers participated in a Delphi study. Results: A lack of knowledge regarding how to best help and having to deal with the student’s problems were identified as difficulties. Specific training, psychological support, and advice from health professionals were the most commonly reported needs. Maintaining contact with the family and the students and providing personalized attention were viewed as the most useful resources. Finally, knowledge about the disease itself and how to facilitate successful school re-entry were identified as important program components. Conclusion: The findings provide important new information regarding the lack of both resources and support for teachers who seek to help youth cancer survivors. The findings can be used to inform the development of an intervention to help teachers become more successful in facilitating successful school re-entry.

## 1. Introduction

Cancer survivors, including children, can experience a number of physical [1,2,3] and psychological symptoms related to their cancer history [4,5,6]. In youths, these symptoms have been shown to be related with measures of school dysfunction [7,8], such as lower grades across subjects [9], school absenteeism and the need to repeat a grade [7], and difficulties related to coming back to school, such as concentration and attentional problems, socialization needs, and fatigue [10]. However, despite the co-morbid symptoms associated with having a history of cancer, we also know that young cancer survivors can have successful lives, particularly if they return to school as soon as it is possible after treatment [11,12,13]. This is not surprising, given that one of the most important needs reported by these children is returning to a normal life [14]. 

The process of returning to school after cancer treatment (“school re-entry”) presents various challenges for the healthcare team, patient, classmates, parents, and teachers [15,16]. For example, teachers report that they worry about their lack of knowledge about cancer and how other children in the classroom will adjust to the cancer survivor child’s return [12,17,18]. Teachers also report having received little training about the school re-entry process and/or may not feel equipped to help this population [12,19], despite the fact that they report a high level of interest in getting guidance on how to help children in the school re-entry process [20]. Given the positive contributions that teachers can have on the quality of life of their students [21], it is important that they acquire the tools and strategies that could best facilitate this. In order to develop teacher education and training programs to address this need, more information regarding the specific difficulties that teachers face regarding school re-entry is needed.

Therefore, the primary objectives of this study were to (1) better understand the difficulties that teachers encounter in helping youth cancer survivors to improve their school experience and (2) to identify the specific needs that teachers have to enhance their ability to contribute to a better school readjustment of these youths and, therefore, help improve their quality of life. As secondary objectives, this study sought to (1) identify the most effective resources, techniques, or strategies that teachers are currently using to help students in the re-entry process and (2) to identify the ideal contents for a program that could help teachers work more effectively with these students. Based on the research summarized previously, we anticipated that teachers would identify a lack of knowledge about the health and psychosocial problems that youth cancer survivors have as a common difficulty, while endorsing a need for specific training for helping students reintegrate into the classroom and assist them to more effectively cope with and adjust to their situation at school. 

## 2. Materials and Methods

### 2.1. Study Design

We used the Delphi method to address the study aims. This method involved identifying a group of suitable qualified experts and then asking them their opinions about topics of interest in successive anonymous rounds. The Delphi method is commonly used to identify consensus opinions and provide the most accurate answers to difficult questions [14,22]. 

### 2.2. Participants

In order to be eligible for the Delphi panel, teachers were required to have or have had cancer survivors in their classrooms. We used the snowball procedure to recruit teacher participants into the study [23]; that is, we first identified teachers who met the inclusion criteria, and these teachers were invited to both participate and to identify other experienced teachers who might be interested in participating. We also notified potential teacher participants via advertising the study through different social networks (i.e., Facebook, Twitter, and a blog) from our research group: https://www.facebook.com/algos.catedra/, accessed on 2 November, 2021. Information about the study was sent via email to potential participants who expressed an initial interest, and a link was forwarded to them which allowed access to the informed consent form and the survey questions. 

### 2.3. Measures and Procedures

Two rounds were needed to achieve consensus. Both rounds took approximately 11 min each to complete.

#### 2.3.1. Round I

Panelists were asked to provide demographic and descriptive data and answer four open-ended questions related to the two primary and two secondary study aims. The open-ended questions asked participants to (1) list the difficulties that cancer survivor students may have in the classroom; (2) list the needs that teachers have when working with these students; (3) list the resources, techniques, or strategies they have used when working with students who are cancer survivors; and (4) indicate what they thought the content should be for a program designed to help teachers work with these students. Panelists were also given the option of making additional comments on the topic of the study. 

#### 2.3.2. Round II

Four lists of items were created from the results of Round I: (1) a list of difficulties; (2) a list of needs; (3) a list of resources, techniques, or strategies; and (4) a list of potential intervention contents. With respect to the list of difficulties, participants were asked to rate the frequency to which each difficulty listed occurred on a 0–4 Likert scale (0 = “Not frequent at all” to 4 = “Extremely frequent”). With respect to the list of needs, participants were asked to rate the extent to which each need they had was currently addressed (0 = “Not addressed at all” to 4 = “Completely addressed”). With respect to the list of resources, techniques, or strategies participants were asked to rate the usefulness of each resource, technique, or strategy (0 = “Not useful at all” to 4 = “Extremely useful”). Finally, participants were asked to rate the importance of potential program content (0 = “Not important at all” to 4 = “Extremely important”). Participants were also given the option of adding new items to each list, if they thought that any of the lists were incomplete. 

### 2.4. Data Analysis

#### 2.4.1. Round I

Demographic and descriptive data from the study participants were summarized using means and ranges for continuous variables and number and percentages for categorical variables. Data from Round I open-ended questions about difficulties, needs, resources, and program contents were qualitatively analyzed and categorized using the constant comparative analysis [24]. 

#### 2.4.2. Round II

We computed the mean ratings associated with each item from the four lists created from the Round I data. At least 75% of the participants needed to respond with a 3 or 4 rating to any one item on the difficulties; resources, techniques, or strategies; and intervention content lists to determine that consensus was achieved for that item. Similarly, 75% of the participants needed to respond with a 0 or 1 to any one item on the needs list in order to determine that there was consensus regarding the need to address a specific concern. This criterion is the median threshold used to define consensus in Delphi studies [25,26]. 

## 3. Results

### 3.1. Round I

Twenty-eight teachers participated in the first Delphi round. The majority (86%) of these were women, teaching at an urban public school at the primary level. Their mean age was 36 years. Additional details regarding the demographic characteristics of the Round 1 participants are summarized in Table 1. 

Table 2 summarizes the results of Round I. Four lists were generated based on the participants’ answers to the Round I open-ended questions asking about (1) classroom difficulties that teachers may have with helping cancer survivor students; (2) teachers’ needs when working with these students; (3) resources, techniques, or strategies that teachers have used to work with these students; and (4) potential contents of a training program that would help teachers work with these students. 

Fifteen items were listed as difficulties. The most commonly reported difficulties were student psychological (reported 14 times) and educational (reported 11 times) difficulties. Eighteen items were listed as needs. The most common of these were mentioned four times each: (1) being trained to help these students return to school life; (2) being educated about the types of cancer that children can have; (3) understanding the benefits and side effects of cancer treatments; and (4) being educated about cancer recurrence. Twenty-one resources were listed, although each was mentioned only one or two times. Finally, 20 specific program contents were listed, with the most common being emotional intelligence (mentioned five times). 

### 3.2. Round II

The demographic characteristics of Round II participants (n = 20) were similar to those from Round I (see Table 3). 

#### Consensus Regarding the Items on the Four Lists Created in Round I

*Difficulties that teachers may have with cancer survivor students in the classroom.* None of the difficulties reported by the teachers reached consensus. In other words, although the teachers reported that they sometimes experienced some of the difficulties listed, none of the difficulties were rated as occurring for 75% or more of the participants.

*Teachers’ needs when working with students who are cancer survivors.* Consensus was achieved with respect to four of the needs listed: (1) the need to provide greater support for teachers; (2) the need for training regarding types of cancer, the effects of cancer treatments, and the chances of cancer recurrence; (3) the need for teachers to be provided with psychological support; and (4) the need for teachers to have access to advice from health professionals. 

*Resources, techniques, or strategies that teachers have used to work with these students.* Consensus was reached with respect to the usefulness of 15 of the resources identified in Round I. Most of the resources, techniques, or strategies used by teachers to work with these students that reached consensus were related to maintaining contact with the family and the students during and after the illness and providing personalized attention to the student (support, reinforcement, motivation, etc.) during the school re-entry process. Adapting games and asking the student to work with helpful classmates was deemed important by 100% of the participants. 

*Content of a training program that would help teachers work with students who are cancer survivors.* Consensus was reached with respect to the importance of 19 of the 20 items identified in Round I; only the item “Using practical cases based on previous experiences” did not reach consensus. The contents that were identified as important included (1) emotional intelligence, (2) group psychology, (3) methods and strategies to help children on a personal and educational level, (4) motivational learning techniques, (5) personal motivation and the improvement of self-esteem, (6) how to reintegrate the child into school: methodologies and resources, (7) how to adapt content to the student’s needs, and (8) encouraging the relationship with classmates during treatment. The mean item scores of each item and the percentage of the Round II Delphi group that rated them as important are presented in Table 4.

## 4. Discussion

A Delphi method was used in this study to better understand both (1) the difficulties teachers have when working with cancer survivor students and (2) what they need to be able to be more effective at maximizing the quality of life of students with a history of cancer. As hypothesized, a lack of knowledge about the health and psychosocial problems that youth cancer survivors have was identified as a difficulty. For example, participants reported not knowing what sort of difficulties the students may have, what support the students may require, and not understanding the disease or the issues faced by survivors and their families. In addition, having to deal with the student’s psychological and educational challenges were commonly reported difficulties. These findings are not surprising, given that cancer survivors are likely to have side and/or late effects of cancer and its treatment that makes it difficult for them to function well in the academic environment [27,28,29]. 

As hypothesized, training for helping students with a history of cancer reintegrate into the classroom was one of the most commonly reported needs. This finding is consistent with previous studies showing that psychosocial problems are related to difficulties in school re-entry among student cancer survivors, and their need for support, both academically and socially [30,31]. Moreover, education about types of cancer and the effects of cancer treatments, the availability of psychological support, and having greater access to advice from health professionals were commonly reported needs. Nevertheless, these needs are not necessarily specific to teachers working with cancer survivor students only, as studies with students that have other medical conditions also report these needs. For example, a qualitative study with primary school teachers who were involved in a school reintegration program for students with burn injuries reported the need to provide support to teachers and the need to develop school reintegration programs [32]. Similarly, in a study of teachers of students with diabetes, teachers were more likely to perceive improvements in students’ self-management when health professionals visited the classroom more often [33].

As secondary objectives to this study, we sought to identify the most effective resources, techniques, and strategies that teachers are currently using in their attempts to help cancer survivor students, as well as the ideal contents of an educational program to help teachers work more effectively with these students. This study showed that maintaining contact with the family and the students and providing personalized attention to the students were the most useful resources. These results are in line with previous research conducted in different student samples. For example, a study with 19 adolescent cancer survivors, 21 mothers, 15 fathers, and 15 siblings estimated the support that adolescents received from friends, teachers, tutors, and the hospital outreach nurse as instrumental in creating a positive school re-entry experience [28]. Similarly, another study conducted with 88 adolescent cancer patients and 40 oncology practitioners rated peer support as the most important factor in education satisfaction for patients [34]. These results, when considered in light of the findings from the current study, stress the importance of developing intervention programs that enhance social support, especially peer support, both during and after cancer treatment.

Regarding the content that these teachers thought should be a part of this program, they indicated, as might be expected, that including content that would help them to address the difficulties and needs that they had previously reported, such as the lack of knowledge about the disease or how to facilitate a successful school re-entry would be helpful to them. Consistent with this idea, researchers have found that increasing knowledge about the disease itself was effective in an education program developed by and for nurses [35]. In this case, the program provided education about social reintegration and school re-entry issues to increase nurses’ self-efficacy in interacting with children, families, teachers, and colleagues. The findings showed a statistically significant increase in teacher knowledge and comfort level when speaking with children and families [35]. In addition, increasing knowledge about cancer might not only help professionals but also classmates. A recent systematic review found that increased knowledge among classmates was associated with less fear and a more positive attitude towards the child with cancer [36]. 

Further support for the potential benefits of training programs also comes from research on the effects of programs designed to help in the re-entry of children with other medical conditions, such as youth with an acquired brain injury. For example, a systematic review collected 17 hospital-to-school reintegration interventions and concluded that cognitive, behavioral, and problem-solving interventions have the potential to improve school reintegration [37]. In the specific case of children with cancer, a comprehensive school reintegration consisting of supportive counseling, educational presentations, systematic liaison between the hospital and the school, and periodic follow-ups was rated as being very useful and of high value by children newly diagnosed with cancer, their parents, and teachers [38]. As a group, these findings indicate that additional research on the effectiveness of reintegration programs for cancer survivors is warranted; the findings from the current study can be used to inform the contents of such programs.

There are a number of limitations of the current study that should be kept in mind when interpreting the results. We used a convenience sample of teachers (the majority were women, teaching at an urban public school). Therefore, the generalizability of the results to other teachers is not known. An important next step would be to replicate this study’s findings in a sample of teachers with different characteristics (e.g., a sample with more men, teachers working in a rural school). In addition, participants had only one cancer survivor student (on average). Future research, ideally with participants with more experience with cancer survivor students, including the hospital school teachers or the psychologists who worked with them, will be needed to determine if those with more experience use more or different helpful resources. Moreover, parental perceptions could be added to understand better the childhood cancer survivors’ needs, as parents are fundamental to help pediatric patients in their re-entry process and they must be considered and helped.

To the best of our knowledge, this is the first study that assesses the problems and needs that teachers have when working with students that are cancer survivors. The current study provides important new information regarding (1) the lack of resources, both at the information/training level, and the lack of support of other professionals that teachers have to face when there are survivors of cancer in the classroom and (2) the relevance of maintaining the student’s social support as a tool for the reincorporation to the classroom. Therefore, future research should develop and test the effectiveness of programs that facilitate school reintegration and improved quality of life. 

## 5. Conclusions

Teachers have an essential role on the re-entry process, as well as in the educational and social reintegration of cancer survivor students. However, this may pose a challenge for many teachers due the difficulties identified in this study. Providing resources to teachers in the re-entry process appears to be very important to helping young cancer survivors return to a successful life. 

## Figures and Tables

**Table 1:** Sample characteristics from Round I (n = 28).

Age mean (SD) and range	36.4 (6.7) 25–50
Sex: women (%)	86
Teaching level, n (%)	
Primary	18 (64.3)
Secondary	8 (28.6)
Others	2 (7.2)
Type of institution, n (%)	
Public	18 (64.3)
Private	8 (28.6)
Concerted	2 (7.1)
Area, n (%)	
Rural	3 (10.7)
Urban	25 (89.3)
Teaching experience, mean years (SD)	9.3 (5.7)
Student cancer survivors, mean (SD)	1.3 (0.5)

**Table 2:** Results from Round I (number of times items were reported).

Difficulties that teachers may have with cancer survivor students in the classroom. Teachers may:	
Have to deal with the student’s physical difficulties: relapse, occasional indisposition, physical discomfort, etc.	4
Have to deal with the student’s psychological difficulties: attention problems, low self-esteem, attitudinal problems, lack of motivation, socialization, depression, anxiety, etc.	14
Have to deal with the student’s difficulties at the educational level: curriculum gap, lack of attendance, different learning rhythm, monitoring of learning, etc.	11
Overprotect the student.	1
Treat the student the same as the others.	1
Work on emotions in the classroom.	1
Work without the (material, human) resources necessary to adequately attend to them.	2
Not know how to talk about what happened.	1
Not know how much they can demand of the student.	1
Not know how the student can adapt to the group.	8
Not know how to treat the student.	2
Not know how to prepare the classmates for the survivor’s return to class.	2
Work with very large numbers of students.	2
Not know what sort of difficulties the student may have.	4
Not know what support these students require.	4
Not understand the disease or the process suffered by the survivors and their families.	1
**Teachers’ needs when working with these students. Teachers must:**	
Know how to work on emotional education.	2
Know how to work on acceptance of the disease.	1
Use group dynamics.	2
Know how to create awareness in the rest of the class group.	1
Encourage family–school collaboration.	2
Use material resources to help the student catch up.	2
Provide the student with extra work and reinforcement.	1
Make greater use of support teachers in the classroom.	2
Use methodologies and tools so that these students can better adapt to the school/institute.	2
Be trained to help these students get back into school life.	4
Be trained in motivational classroom techniques and resources.	2
Be trained in the physical and mental health of this type of student.	2
Be trained in relaxation and meditation techniques.	2
Be trained in the needs of these students during and after treatment.	2
Be trained in types of cancer, effects of treatments, and recurrences.	4
Be provided with psychological support.	1
Be advised by health professionals.	3
Be provided with updated information on the student’s physical and mental condition.	3
**Resources, techniques, or strategies that teachers have used to work with these students. Teachers have:**	
Used emotional education.	1
Used common sense.	1
Been trained in social skills to promote their reintegration.	1
Made the student’s classmates aware of the disease.	1
Given the student the opportunity to talk about their experience so that everyone can understand him/her.	1
Given personalized attention so that they can complete their tasks.	2
Given them reinforcement and extra help.	1
Asked the student to sit in the front row.	1
Motivated and praised him/her when they do well.	1
Asked the student to work with helpful classmates.	2
Not drawn attention to the student in front of others if the student is disruptive.	1
Observed the student (to see if he/she is distracted, sleepy, or uncomfortable).	2
Given the student support and conversation.	1
Used new technologies to monitor and deliver work.	1
Treated the student normally, although without forgetting their situation.	2
Worked in a fun and motivating way.	1
Adapted games so that they can take part.	1
Kept in touch with the student throughout his/her illness.	1
Kept in touch with the family throughout the illness.	2
Kept in touch with the family after the illness.	2
Entered into agreements and work commitments with the educational team, families, and the student.	1
**Content of a training program that will help teachers work with these students**	
Emotional intelligence.	5
Group psychology.	2
Psychological support for the student.	2
Attention to diversity.	1
Effects of cancer treatments on a physical and psychological level.	2
Problems of cancer survivor students. Knowledge on what life is like after this disease. Cancer detection, treatment, and follow up.	3
Individualized information on what the healing process has been like, the time it has taken to heal, current medication and habits, and what the student has been taught in hospital (if there were hospital classes).	1
Counseling by the medical staff and by parents about the needs of the student.	1
Methods and strategies to help children on a personal and educational level.	2
Motivational learning techniques.	2
Personal motivation and the improvement of self-esteem.	1
Practical cases.	1
Experiences of other teachers.	1
How to help families.	2
How to reintegrate the child into school: methodologies and resources.	3
How to adapt content to the student’s needs.	3
Awareness of how to work with the rest of the class.	2
Awareness and preparation of the school for the return of the student.	1
Explaining the situation to their classmates.	1
Encouraging the relationship with classmates during treatment.	2

**Table 3:** Sample characteristics from Round II (n = 20).

Age mean (SD) and range:	37.6 (8.1) 27–57
Sex: women (%)	80
Teaching level, n (%)	
Primary	11 (55)
Secondary	6 (30)
Others	3 (15)
Type of institution, n (%)	
Public	15 (75)
Private	1 (5)
Concerted	4 (20)
Area, n (%)	
Rural	4 (20)
Urban	16 (80)
Teaching experience, mean years (SD)	11.1 (8.7)
Student cancer survivors, mean (SD)	1.4 (0.6)

**Table 4:** Results from Round II.

Difficulties that teachers may have with cancer survivor students in the classroom. Teachers may:	Mean SD	Consensus (%)
Have to deal with the student’s physical difficulties: relapse, occasional indisposition, physical discomfort, etc.	2.7→1.1	
Have to deal with the student’s psychological difficulties: attention problems, low self-esteem, attitudinal problems, lack of motivation, socialization, depression, anxiety, etc.	2.6→1.0	
Have to deal with the student’s difficulties at the educational level: curriculum gap, lack of attendance, different learning rhythm, monitoring of learning, etc.	2.4→1.1	
Overprotect the student.	1.7→1.2	
Treat the student the same as the others.	2.3→1.4	
Work on emotions in the classroom.	2.6→1.3	
Work without the (material, human) resources necessary to adequately attend to them.	2.5→1.4	
Not know how to talk about what happened.	2.5→1.1	
Not know how much they can demand of the student.	2.3→1.3	
Not know how the student can adapt to the group.	1.9→1.3	
Not know how to treat the student.	1.7→1.2	
Not know how to prepare the classmates for the survivor’s return to class.	1.9→1.2	
Work with very large numbers of students.	2.8→1.4	
Not know what sort of difficulties the student may have.	2.2→0.9	
Not know what support these students require	2.3→1.0	
Not understand the disease or the process suffered by the survivors and their families.	1.9→1.0	
**Teachers’ needs when working with these students. Teachers must:**		
Know how to work on emotional education.	2.0→1.0	
Know how to work on acceptance of the disease.	1.4→0.9	
Use group dynamics.	1.8→1.3	
Know how to create awareness in the rest of the class group.	1.8→1.2	
Encourage family–school collaboration.	2.5→0.9	
Use material resources to help the student catch up.	2.5→0.9	
Provide the student with extra work and reinforcement.	2.7→1.0	
Make greater use of support teachers in the classroom.	0.8→1.0	75
Use methodologies and tools so that these students can better adapt to the school/institute.	2.3→1.1	
Be trained to help these students get back into school life.	1.7→0.8	
Be trained in motivational classroom techniques and resources.	2.2→1.1	
Be trained in the physical and mental health of this type of student.	1.2→1.1	
Be trained in relaxation and meditation techniques.	1.3→1.2	
Be trained in the needs of these students during and after treatment.	1.2→1.2	
Be trained in types of cancer, effects of treatments, and recurrences.	1.0→1.0	75
Be provided with psychological support.	0.7→1.2	85
Be advised by health professionals.	0.8→1.1	80
Be provided with updated information on the student’s physical and mental condition.	1.5→1.4	
**Resources, techniques, or strategies that teachers have used to work with these students. Teachers have:**		
Used emotional education.	3.4→0.8	80
Used common sense.	3.5→0.7	90
Been trained in social skills to promote their reintegration.	3.1→1.0	
Made the student’s classmates aware of the disease.	2.9→1.2	
Given the student the opportunity to talk about their experience so that everyone can understand him/her.	2.9→1.1	
Given personalized attention so that they can complete their tasks.	3.2→0.8	80
Given them reinforcement and extra help.	3.3→0.7	85
Asked the student to sit in the front row.	2.1→1.2	
Motivated and praised him/her when they do well.	3.0→0.6	90
Asked the student to work with helpful classmates.	3.4→0.5	100
Not drawn attention to the student in front of others if the student is disruptive.	1.7→1.2	
Observed the student (to see if he/she is distracted, sleepy, or uncomfortable).	3.4→0.7	90
Given the student support and conversation.	3.7→0.5	95
Used new technologies to monitor and deliver work.	2.8→1.3	
Treated the student normally, although without forgetting their situation.	3.3→0.7	85
Worked in a fun and motivating way.	3.5→0.6	95
Adapted games so that they can take part.	3.5→0.5	100
Kept in touch with the student throughout his/her illness.	3.5→0.6	90
Kept in touch with the family throughout the illness.	3.5→0.7	90
Kept in touch with the family after the illness.	3.4→0.7	85
Entered into agreements and work commitments with the educational team, families, and the student.	3.3→0.7	85
**Content of a training program that will help teachers work with these students**		
Emotional intelligence.	3.9→0.3	100
Group psychology.	3.6→0.5	100
Psychological support for the student.	3.7→0.5	95
Attention to diversity.	3.4→0.9	80
Effects of cancer treatments on a physical and psychological level.	3.7→0.6	95
Individualized information on what the healing process has been like, the time it has taken to heal, current medication and habits, and what the student has been taught in hospital (if there were hospital classes).	3.2→0.9	75
Counseling by the medical staff and by parents about the needs of the student.	3.5→0.6	95
Methods and strategies to help children on a personal and educational level.	3.7→0.4	100
Motivational learning techniques.	3.6→0.5	100
Personal motivation and the improvement of self-esteem.	3.7→0.4	100
Practical cases.	3.3→0.8	
Experiences of other teachers.	3.6→0.6	95
How to help families.	3.7→0.5	95
How to reintegrate the child into school: methodologies and resources.	3.8→0.4	100
How to adapt content to the student’s needs.	3.6→0.5	100
Awareness of how to work with the rest of the class.	3.6→0.6	90
Awareness and preparation of the school for the return of the student.	3.6→0.7	90
Explaining the situation to their classmates.	3.7→0.6	90
Encouraging the relationship with classmates during treatment.	3.7→0.4	100

## Data Availability

The dataset used and analyzed in this study is available from the corresponding author upon request.
